# Supplementary material for: Analysis of the Content and Comprehensiveness of Dermatology Residency Training Websites in Taiwan
Source: Healthcare (Basel). 2021 Jun 21;9(6):773. doi: 10.3390/healthcare9060773 (PMC8235558; doi:10.3390/healthcare9060773)
Supplement: Supplementary file 1 [file healthcare-09-00773-s001.zip › healthcare-1255640-supplementary.pdf]

Supplementary materials

Supplement. Table S1. Dermatology residency websites included in this study.

Name of Hospital	Public/Private	Website Analyzed
Northern Area (13) *		
Keelung Chang Gung Memorial Hospital and Lovers Lake Branch	Private	https://www.cgmh.org.tw/tw/Systems/BranchInfo/2/XXXXX/23800 (accessed on 20, August, 2020; 22, September, 2020)
Taipei Veterans General Hospital	Public	https://wd.vghtpe.gov.tw/derm/Index.action(accessed on 20, August, 2020; 22, September, 2020)
National Taiwan University Hospital	Public	https://www.ntuh.gov.tw/derm/Index.action(accessed on 20, August, 2020; 22, September, 2020)
Tri-service General Hospital	Public	https://wwwv.tsgh.ndmctsgh.edu.tw/unit/10016/13279(accessed on 20, August, 2020; 22, September, 2020)
Mackay Memorial Hospital	Private	http://www.mmh.org.tw/taitam/derma/index-new.html(accessed on 20, August, 2020; 22, September, 2020)
Taipei Municipal Wangfang Hospital†	Public	https://www.wanfang.gov.tw/p9_medical.aspx?dn = %E5%85%B6%E4%BB%96%E5%B0%88%E7%A7%91&cn = %E7%9A%AE%E8%86%9A%E7%A7%91(accessed on 20, August, 2020; 22, September, 2020)
Taipei Medical University Hospital	Private	https://www.tmuh.org.tw/team/team/11/215
Shin Kong Wu Ho Su Memorial Hospital	Private	https://www.skh.org.tw/skh/d89e980b15.html(accessed on 20, August, 2020; 22, September, 2020)
Cathay General Hospital	Private	https://www.cgh.org.tw/ec99/rwd1320/category.asp?category_id=452
Taipei City Hospital, Renai Branch and Heping Fuyou Branch	Public	https://tpech.gov.taipei/mp109161/cp.aspx?n = 66FFD9FE848887A3&s=FF8E314F302FACC6 https://tpech.gov.taipei/mp109151/cp.aspx?n = D8CE035C19837302(accessed on 20, August, 2020; 22, September, 2020)
Taipei and Linkou Chang Gung Memorial Hospital	Private	https://www1.cgmh.org.tw/branch/lnk/2016/dept2.aspx?deptId=33800 (accessed on 20, August, 2020; 22, September, 2020)

		https://www.cgmh.org.tw/tw/Systems/BranchInfo/1/XXXXX/13800(accessed on 20, August, 2020; 22, September, 2020)
Far Eastern Memorial Hospital	Private	https://www.femh.org.tw/section/Secbrief.aspx?CID=0240&ReturnUrl=https://bald-headed-wayside.000webhostapp.com/(accessed on 20, August, 2020; 22, September, 2020)
Shuang Ho Hospital, Ministry of Health and Welfare†	Public	https://shh.tmu.edu.tw/page/ShhregTable.aspx?deptCode=11(accessed on 20, August, 2020; 22, September, 2020)
Central Area (4) *		
Taichung Veterans General Hospital	Public	http://www.vghtc.gov.tw/(X(1)S(gueeqnmmyiwccltmdtrzblyn))/PageView/UnitTemplate1?UnitID=7cda4673-6096-4748-a034-3bab9638288c&UnitDefaultTemplate=1(accessed on 20, August, 2020; 22, September, 2020)
China Medical University Hospital	Private	https://www.cmuh.cmu.edu.tw/Department/Detail?depid=77(accessed on 20, August, 2020; 22, September, 2020)
Chung Shan Medical University Hospital	Private	http://web.csh.org.tw/web/a29010/(accessed on 20, August, 2020; 22, September, 2020)
Changhua Christian Hospital	Private	http://www2.cch.org.tw/layout_5/about.aspx?id=800(accessed on 20, August, 2020; 22, September, 2020)
Southern Area (5) *		
National Cheng Kung University Hospital	Public	http://derm.med.ncku.edu.tw/index.php(accessed on 20, August, 2020; 22, September, 2020)
Chi Mei Medical Center	Private	http://www.chimei.org.tw/main/cmh_department/top/57770_index.html(accessed on 20, August, 2020; 22, September, 2020)
Kaohsiung Medical University Chung-Ho Memorial Hospital	Private	http://www2.kmuh.org.tw/web/kmuhdept/0900/DeptIntro.aspx(accessed on 20, August, 2020; 22, September, 2020)
Kaohsiung Veterans General Hospital	Public	https://org.vghks.gov.tw/derm/Default.aspx?r=73608461(accessed on 20, August, 2020; 22, September, 2020)
Kaohsiung Chang Gung Memorial Hospital	Private	https://www.cgmh.org.tw/tw/Systems/BranchInfo/8/XXXXX/83800(a

		ccessed on 20, August, 2020; 22, September, 2020)
Eastern Area (1) *		
Hualien Tzu Chi Hospital	Private	https://hlm.tzuchi.com.tw/dermat/ (accessed on 20, August, 2020; 22, September, 2020)

*The hospitals were divided into four areas (i.e., northern, central, southern and eastern), according to location, Council for Economic Planning and Development, Executive Yuan. †The Taipei Municipal Wangfang Hospital; Shuang Ho Hospital, Ministry of Health and Welfare were regarded as public hospitals even if these were managed by the Taipei Medical University.
